# Supplementary material for: Topical tacrolimus for the treatment of secondary lymphedema
Source: Nat Commun. 2017 Feb 10;8:14345. doi: 10.1038/ncomms14345 (PMC5309859; doi:10.1038/ncomms14345)
Supplement: Supplementary Information — Supplementary Figures. [file ncomms14345-s1.pdf]

Supplementary Figures

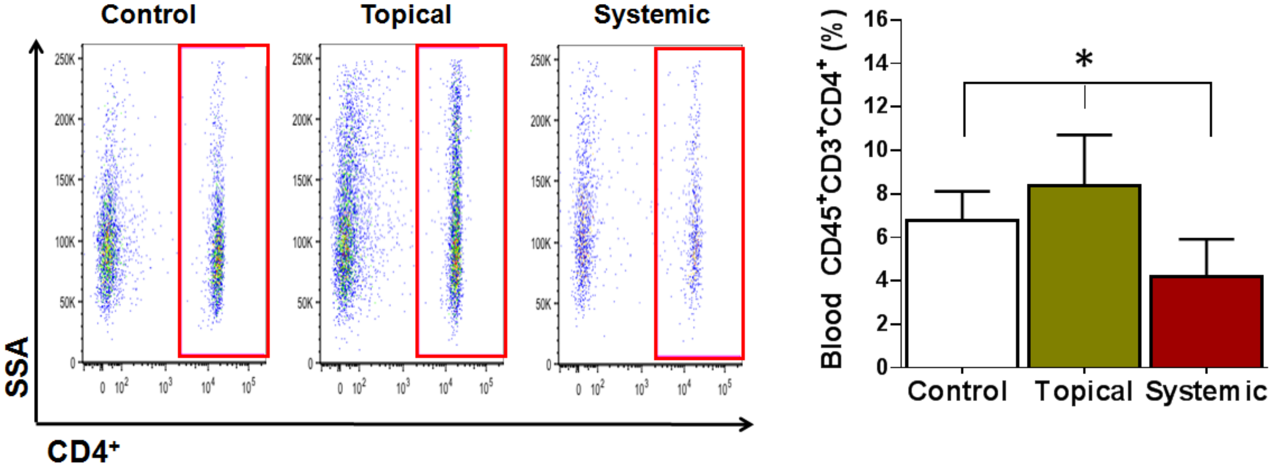

Supplementary Fig. 1: Topical Tacrolimus does not decrease circulating $CD4^+$ T cells. Representative flow plots of peripheral blood $CD4^+$ cells (left panel) with quantification of $CD4^+$ T cells (right-bar graph) shown after 2 week treatment with topical tacrolimus, systemic tacrolimus, or vehicle control. (n=6/group). Experiments were repeated 2 times. All data represent mean ± s.d with $p \leq 0.05$ considered as significant. Data analyzed by ANOVA with post hoc tests.

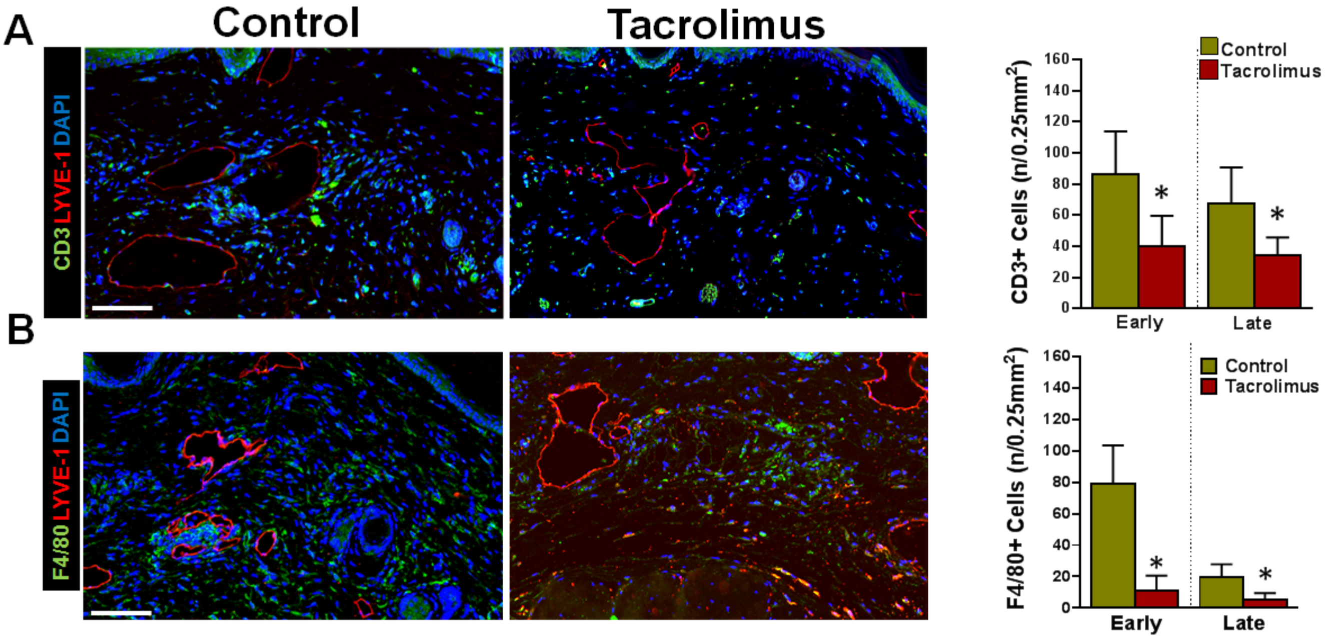

Supplementary Fig. 2: Topical tacrolimus decreases T cell and macrophage infiltration in post-surgical lymphedema.

A. Representative 40x images of tail tissue sections from control and early topical tacrolimus treated animals harvested 6 weeks after surgery with immunofluorescent localization of $CD3^+$ cells (green). Quantification for both early and late treatment experiments is shown to the right (n=6/group). **B**. Representative 40x images of tail tissue sections from control and early topical tacrolimus treated animals harvested 6 weeks after surgery with immunofluorescent localization of $F4/80^+$ cells (green). Quantification for both early and late treatment experiments is shown to the right (n=6/group). All experiments were repeated 2 times. All data represent mean ± s.d with p≤0.05 considered as significant. Data analyzed by 2-tailed student's t-Test. Scale=100μm.

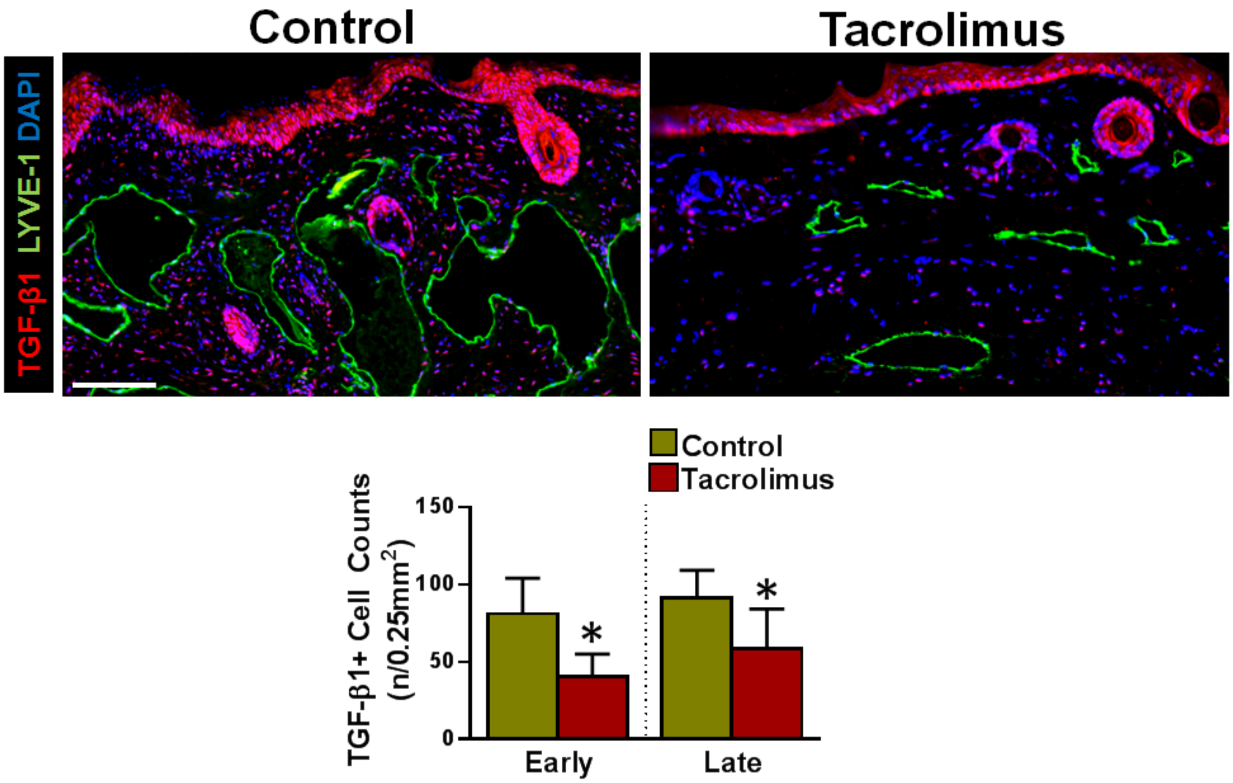

Supplementary Fig. 3: Topical tacrolimus decreases infiltration of TGF-β1 producing cells in post-surgical lymphedema. Representative 40x images of tail tissue sections from control and early topical tacrolimus treated animals harvested 6 weeks after surgery with immunofluorescent localization of TGF-β1^{+} cells (red). Quantification for both early and late treatment experiments is shown below (n=6/group). All experiments were repeated 2 times. All data represent mean ± s.d with $p \leq 0.05$ considered as significant. Data analyzed by 2-tailed student's t-Test. Scale=100μm.

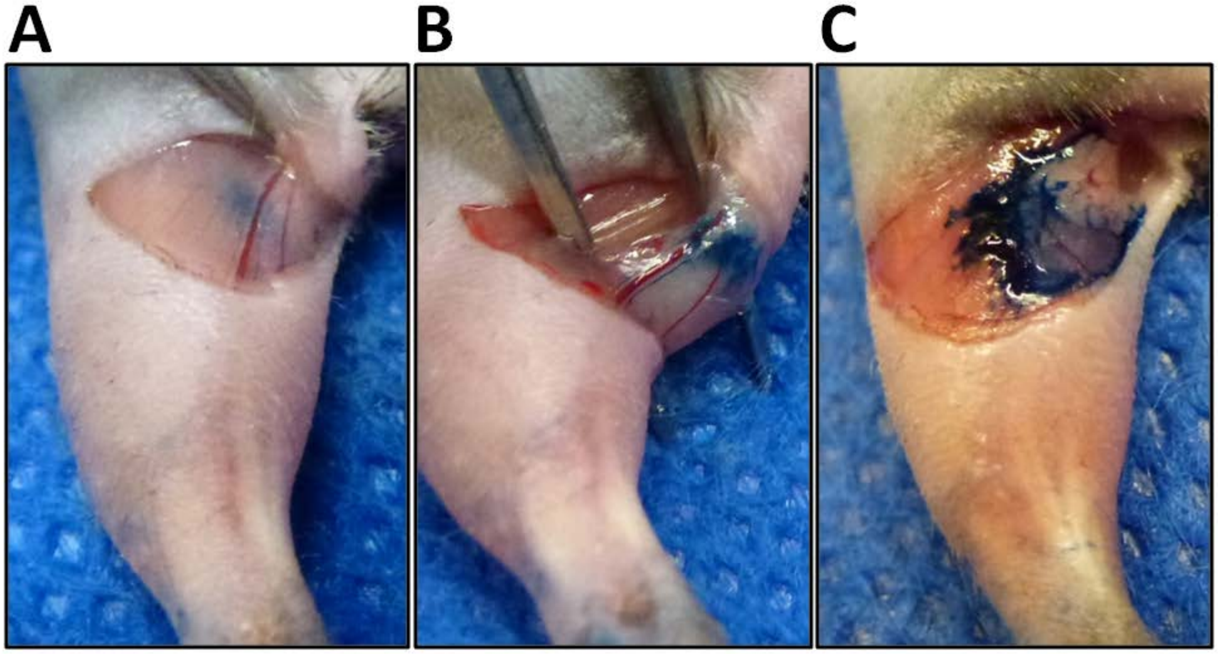

Supplementary Fig. 4: Popliteal lymph node dissection model.

A. The popliteal lymph node, filled with Evan's Blue dye, is visible in the popliteal fat pad.

B. The popliteal lymph node together with afferent and efferent collectors is isolated with its surrounding fat pad.

C. Following surgical resection Evans blue dye is seen spilling freely around the surgical site.

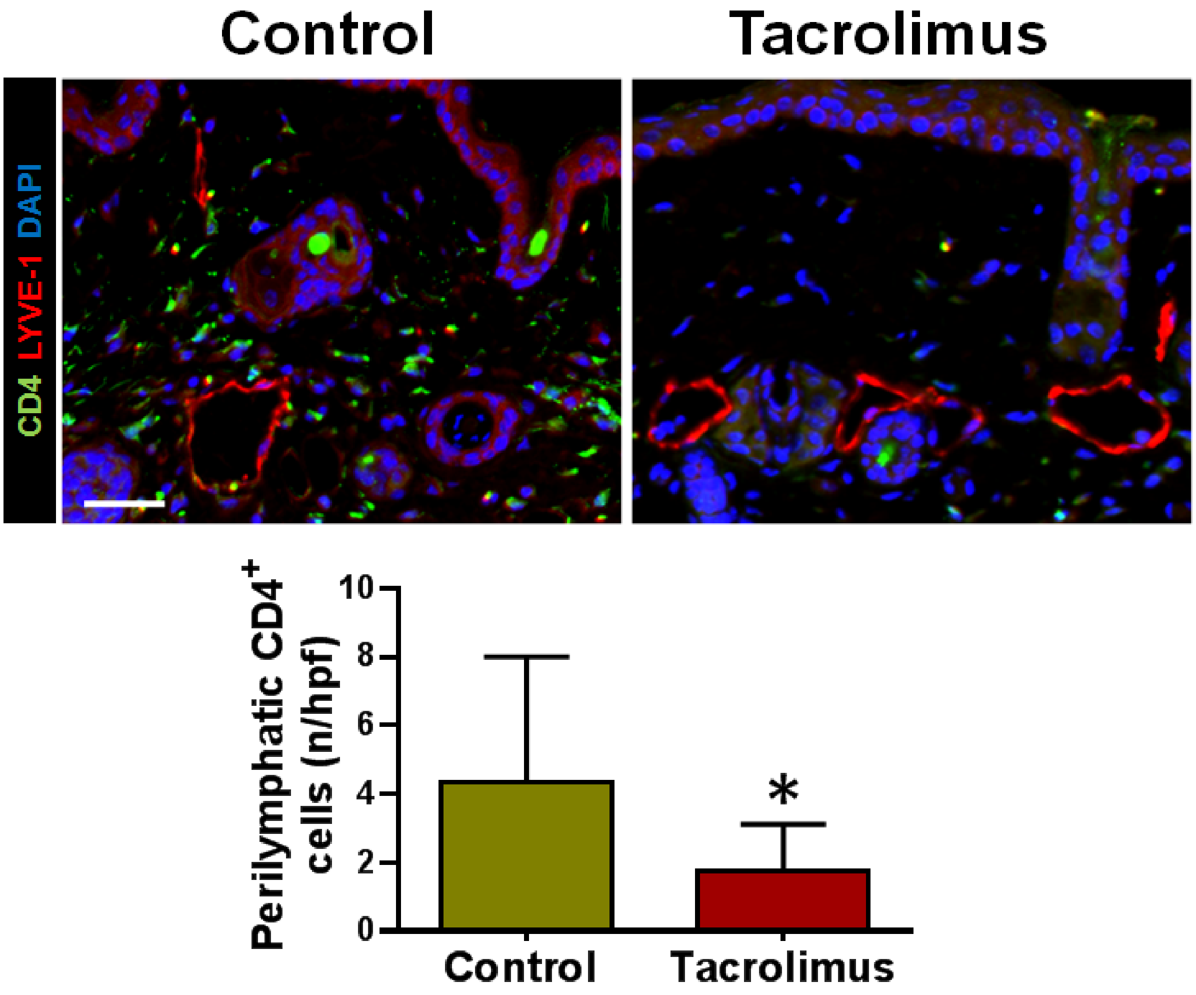

Supplementary Fig. 5: Topical tacrolimus decreases perilymphatic CD4$^+$ cell infiltration after PLND. Representative images of immunofluorescent localization of CD4$^+$ cells (green) and lymphatic vessels (LYVE-1$^+$ red) in animals treated with control or tacrolimus and harvested 4 weeks after PLND. Quantification is shown below (n=6/group). All experiments were repeated 2 times. All data represent mean ± s.d with $p \leq 0.05$ considered as significant. Data analyzed by 2-tailed student's t-Test. Scale=50μm.

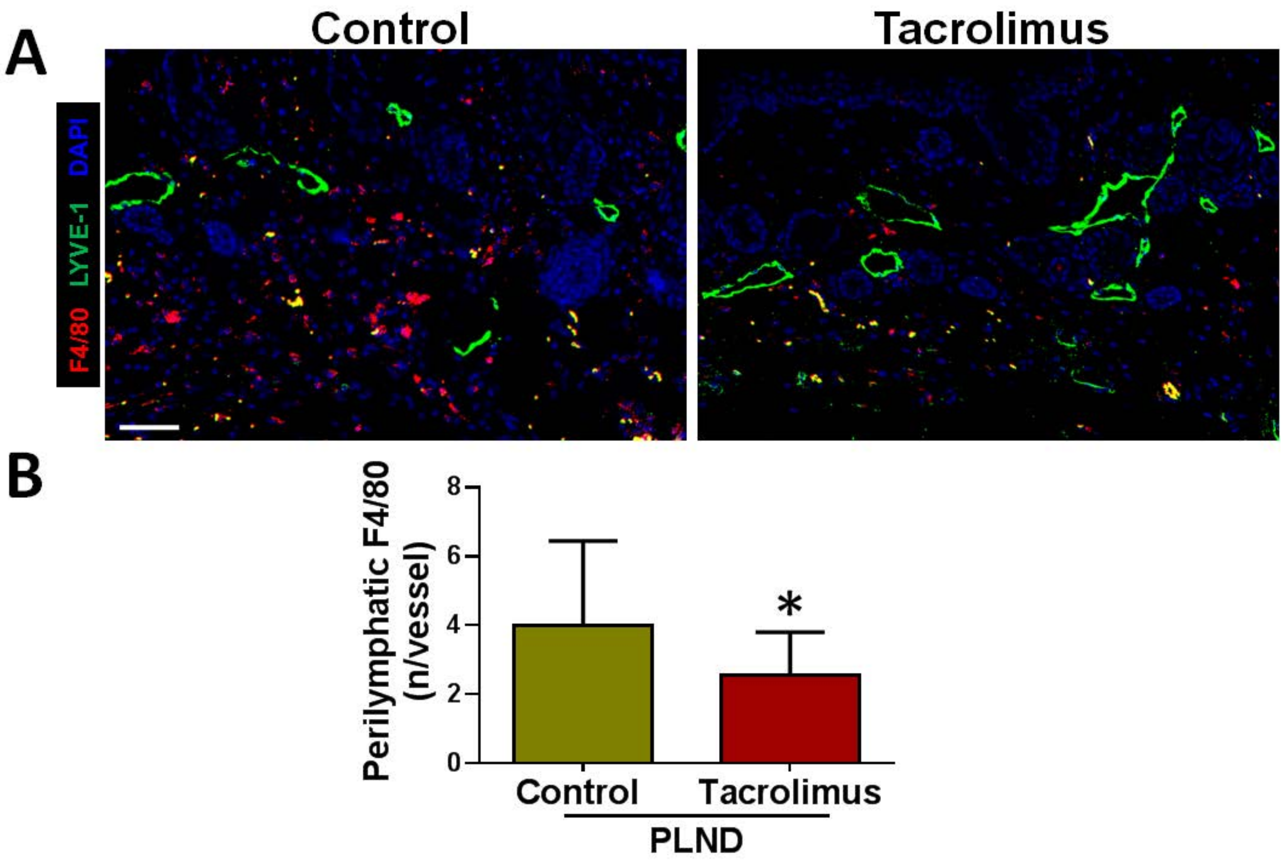

Supplementary Fig. 6: Topical tacrolimus decreases perilymphatic F4/80⁺ cell infiltration after PLND.

A. Representative of immunofluorescent images of tacrolimus and vehicle treated PLND hindlimb skin tissues sections stained for lymphatic vessels (green) and macrophages (red).

B. Quantification of the perilymphatic F4/80+ macrophages (n=6/group). All experiments were repeated 2 times. All data represent mean ± s.d with $p \leq 0.05$ considered as significant. Data analyzed by 2-tailed student's t-Test. Scale=50μm.

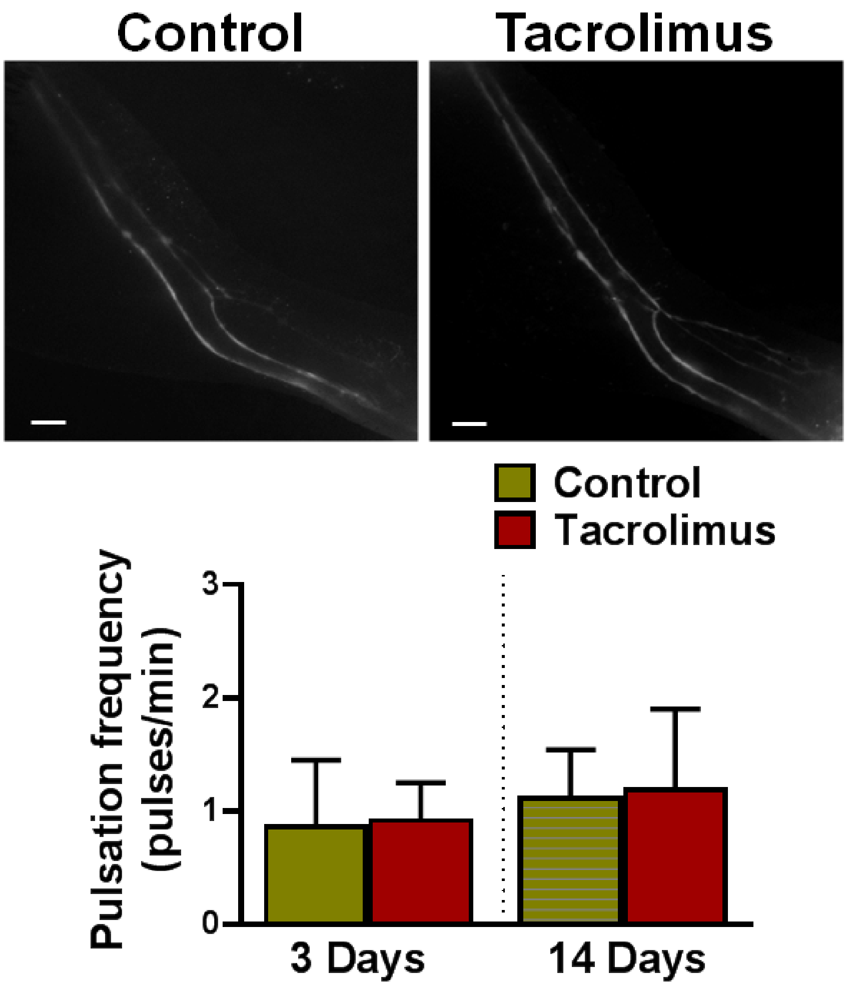

Supplementary Fig. 7: Topical tacrolimus does not alter collecting lymphatic vessel pulsation or lymphangiogenesis in the absence of lymphatic injury or inflammation.

Representative images of NIR lymphatic images of the hind limb lymphatics in sham operated mice (i.e. anesthesia without incision or PLND) following treatment with control or topical tacrolimus for 2 weeks. Quantification of collecting lymphatic vessel pulsation frequency following 3 or 14 days of treatment with tacrolimus is shown below. Video of pulsations can be found in Supplementary movie 2 (n=6/group). All experiments were repeated 2 times. All data represent mean ± s.d with $p \leq 0.05$ considered as significant. Data analyzed by 2-tailed student's t-Test. Scale=1mm.

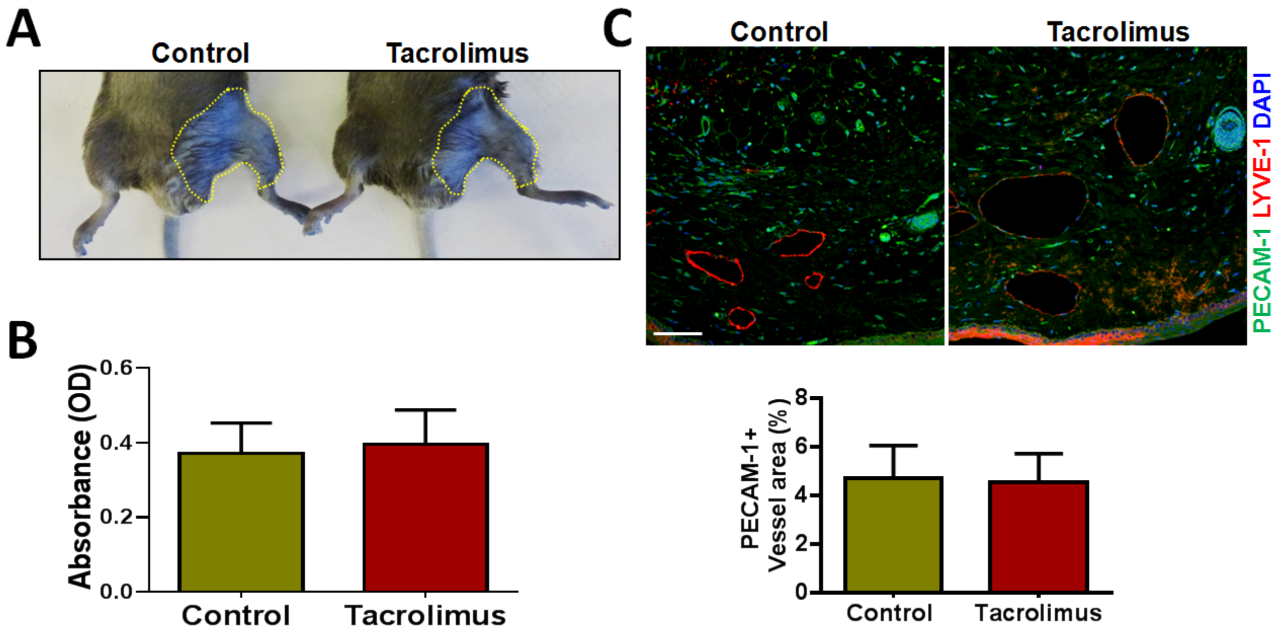

Supplementary Fig. 8: Topical tacrolimus treatment does not alter vascular permeability or vascular density following lymphatic injury.

A. Representative gross images of tacrolimus or vehicle treated PLND mice hindlimbs after tail vein Evans blue injections to measure vascular permeability.

B. Quantification of absorbance of formamide extracted Evans blue from tacrolimus and vehicle treated PLND hind limb tissues.

C. Representative confocal images of tacrolimus or vehicle treated tail skin sections stained for blood vessel marker PECAM-1(green) and the quantification for blood vessel density below (n=6/group). All experiments were repeated 2 times. All data represent mean ± s.d with $p \leq 0.05$ considered as significant. Data analyzed by 2-tailed student's t-Test. Scale=100μm.

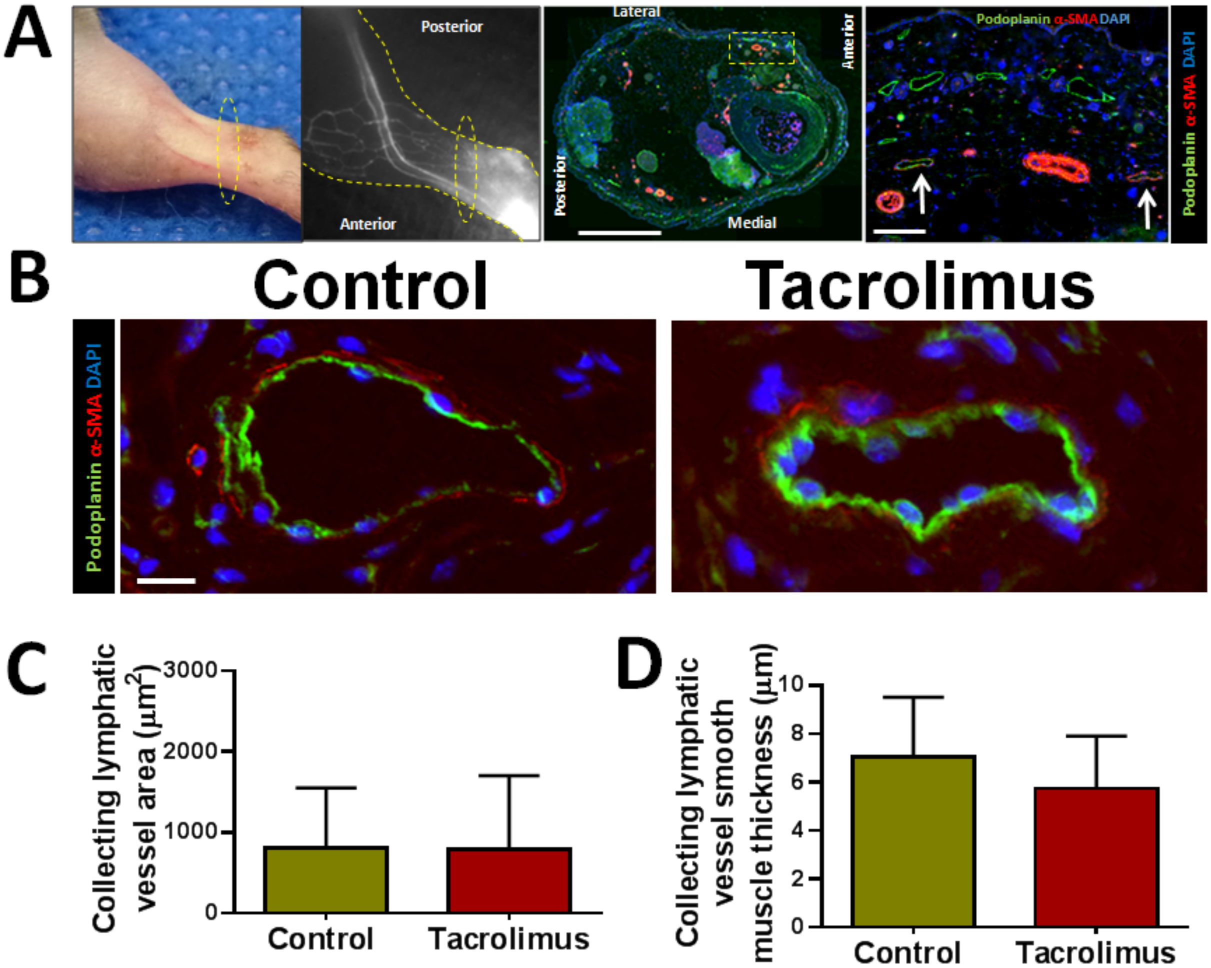

Supplementary Fig. 9: Topical tacrolimus after PLND does not alter α-SMA coverage or luminal diameter of hind limb lymphatic collectors.

A. (Images from left to right) Brightfield image of the lateral aspect of a mouse hind limb with the level of the cross-sections shown by the yellow ellipse. NIR image of a mouse hind limb showing the anatomy of lymphatic vessels of the hind limb, with two large-caliber vessels on the lateral aspect. A 5x IF image of a mouse hind limb with a yellow box indicating the anterolateral leg, where the two dominant collecting lymphatic vessels are located. A 20x image of the region in which the dominant collecting vessels are located (shown with white arrows). Scale= 1mm (middle), 100μm (far right).

B. Representative 100x images of cross-sections of the collecting lymphatic vessels after dual immunofluorescent staining for Podoplanin (Green) and α-SMA (Red). Scale=10μm.

C. Quantification of luminal area.

D. Quantification of the thickness of α-SMA (n=6/group). All experiments were repeated 2 times. All data represent mean ± s.d with $p \leq 0.05$ considered as significant. Data analyzed by 2-tailed student's t-Test.

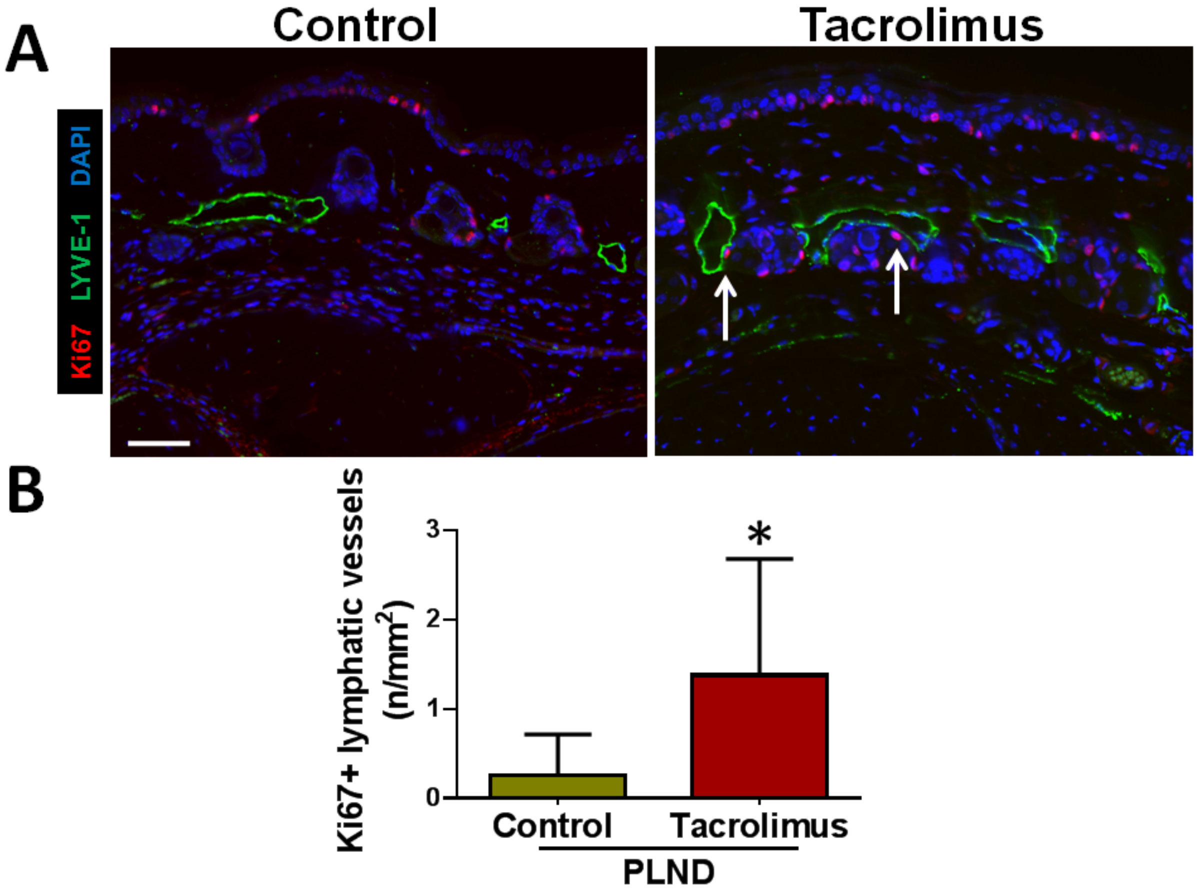

Supplementary Fig. 10: Topical tacrolimus increases Ki67+ cells in lymphatic vessels in the setting of lymphatic injury.

A. Representative of immunofluorescent images of tacrolimus and vehicle treated PLND hindlimb skin tissues sections stained for lymphatic vessels (green) and a marker of proliferation (Ki67; red). Ki67+ cells in lymphatic vessel walls are indicated with white arrows.

B. Quantification of the percentage of lymphatic vessels expressing Ki67 positivity (n=6/group). All experiments were repeated 2 times. All data represent mean ± s.d with $p \leq 0.05$ considered as significant. Data analyzed by 2-tailed student's t-Test. Scale=50μm

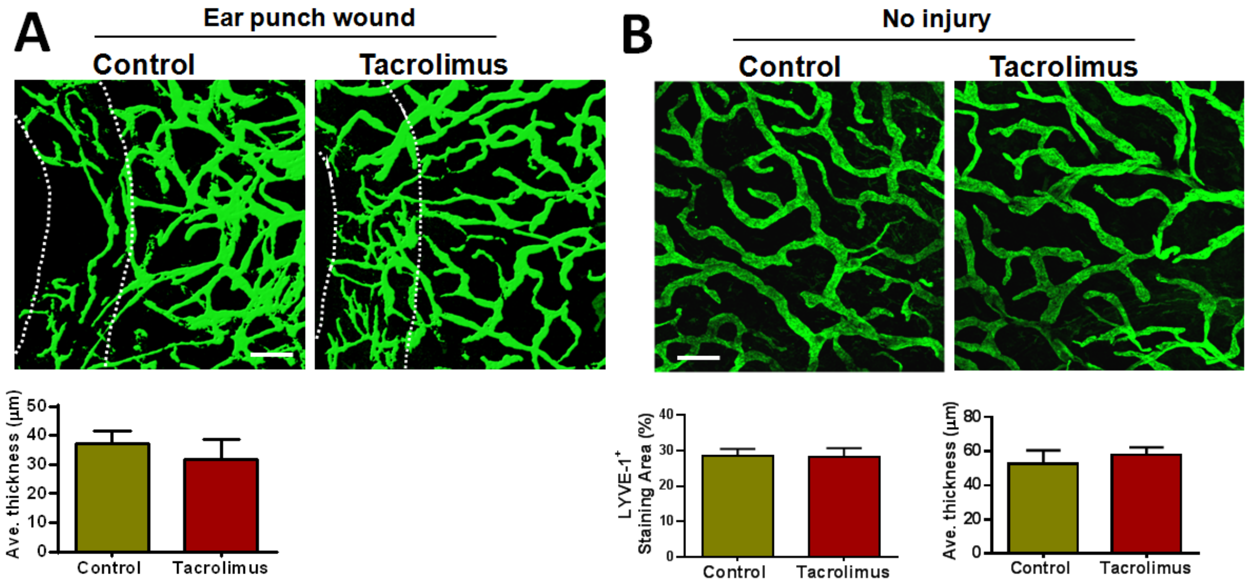

Supplementary Fig. 11: Topical tacrolimus has no effect on lymphatic vessel morphology during injury and lymphatic vessel density in absence of injury respectively

A. Representative surface rendered confocal images of immunofluorescent staining of lymphatic vessels (LYVE-1; green) in punch wound ear skin after 4 weeks of application of topical tacrolimus or vehicle control. Quantification of lymphatic vessel thickness 400 µm around the wound margin shown below.

B. Representative confocal images of immunofluorescent staining of lymphatic vessels (LYVE-1; green) in unwounded mouse ears after 4 weeks of application of topical tacrolimus or vehicle control. Quantification of the LYVE-1^+ staining area and vessel thickness is shown to below. (n=6/group). All experiments were repeated 2 times. All data represent mean ± s.d with $p \leq 0.05$ considered as significant. Data analyzed by 2-tailed student's t-Test. Scale=200 µm.

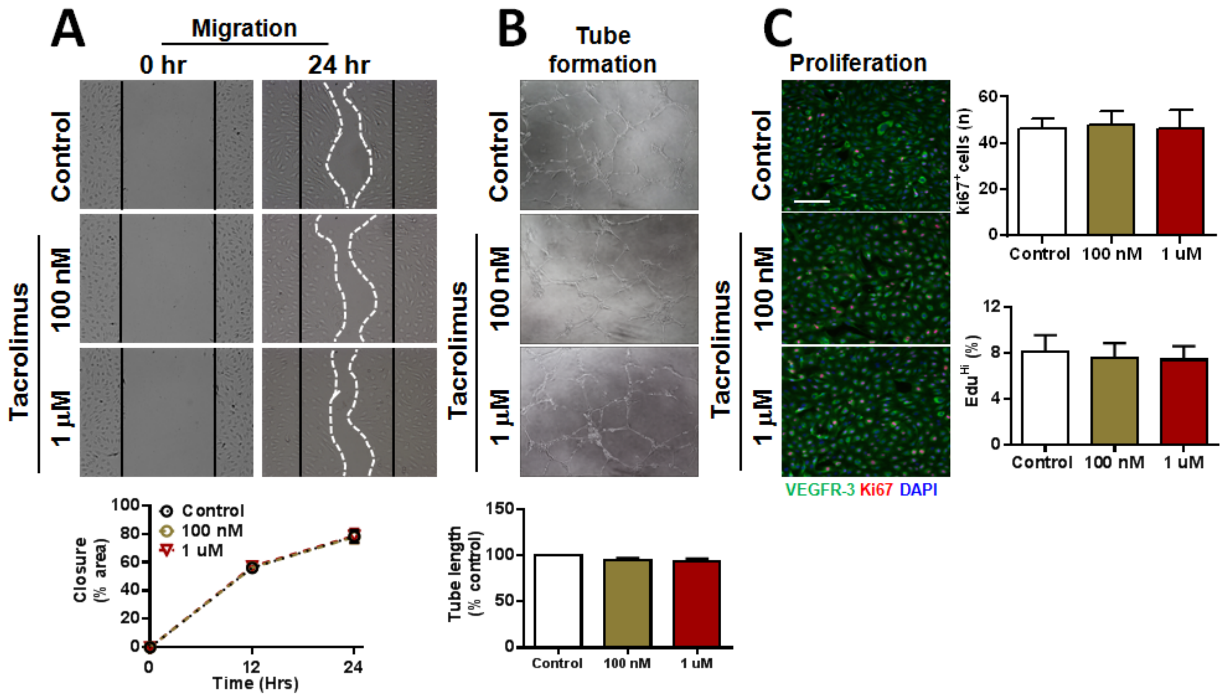

Supplementary Fig. 12: Tacrolimus has no direct effect on LECs migration, tube formation and proliferation.

A. Representative phase contrast images of control and tacrolimus treated LECs, at 0 hr and 24 hr after scratch wound. Quantification of the wound closure below.

B. Representative phase contrast images of control and tacrolimus treated LECs tubules on matrigel 24 hr after seeding. Quantification of the tube length below.

C. Representative confocal images of control or tacrolimus treated LECs monolayers stained for proliferation marker (Ki67; red) LECs (VEGFR3; green). Quantification of the Ki67+ cells (top). Bar graph showing the flow cytometry analyzed Edu+ cells from control or tacrolimus treated LECs (Below). (n=6/group). All experiments were repeated 2 times. All data represent mean ± s.d with p≤0.05 considered as significant. Data analyzed by 2-tailed student's t-Test. Scale=100 µm.
